# Supplementary material for: First Isolation and Phylogenetic Analyses of Tick-Borne Encephalitis Virus in Lower Saxony, Germany
Source: Viruses. 2019 May 21;11(5):462. doi: 10.3390/v11050462 (PMC6563265; doi:10.3390/v11050462)
Supplement: Supplementary file 1 [file viruses-11-00462-s001.zip › Supplementary Figures .pptx]

## Slide 1
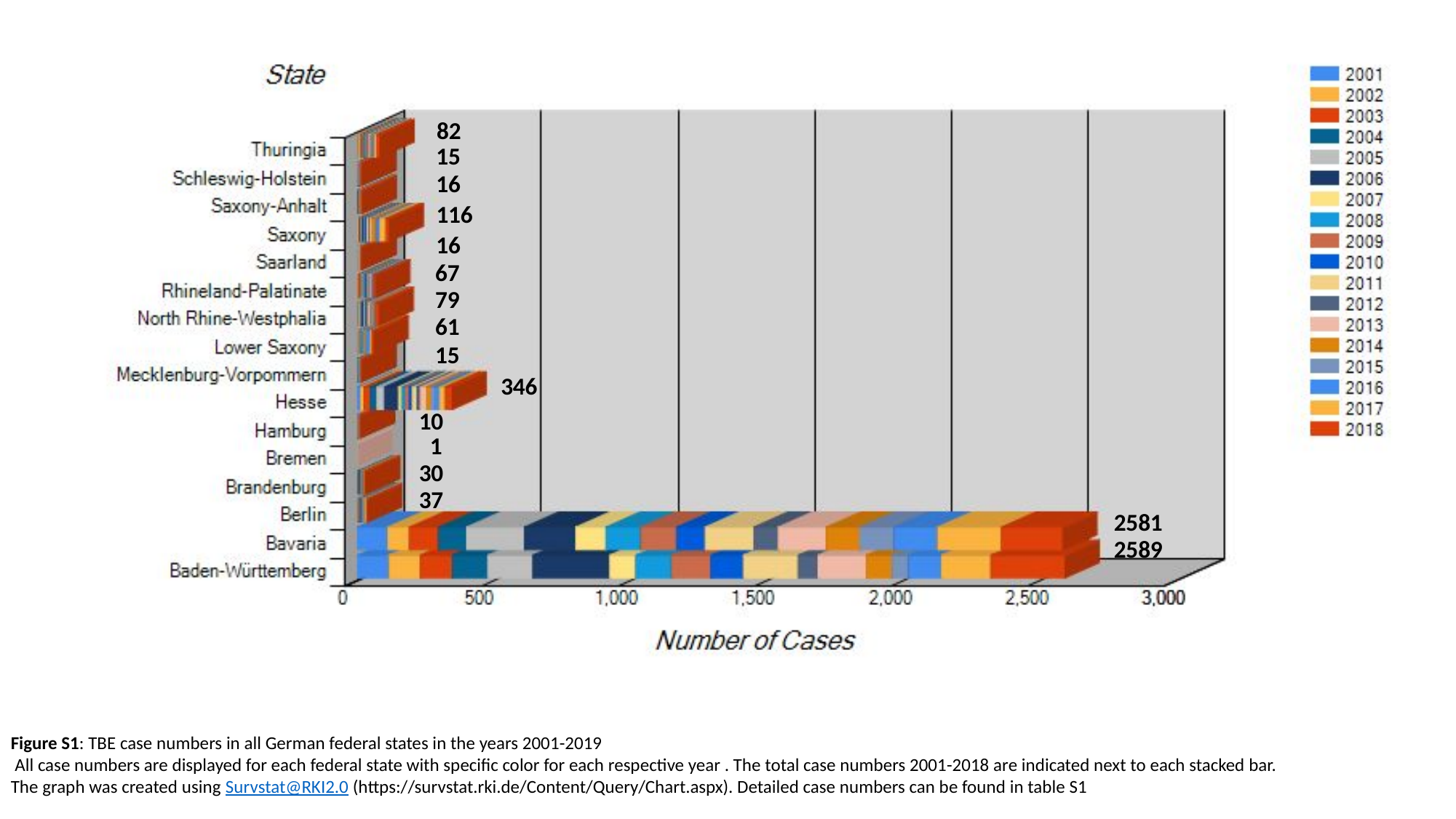

82
15
16
116
16
67
79
61
15
346
10
 1
30
37
2581
2589
Figure S1: TBE case numbers in all German federal states in the years 2001-2019
 All case numbers are displayed for each federal state with specific color for each respective year . The total case numbers 2001-2018 are indicated next to each stacked bar.
The graph was created using Survstat@RKI2.0 (https://survstat.rki.de/Content/Query/Chart.aspx). Detailed case numbers can be found in table S1

## Slide 2
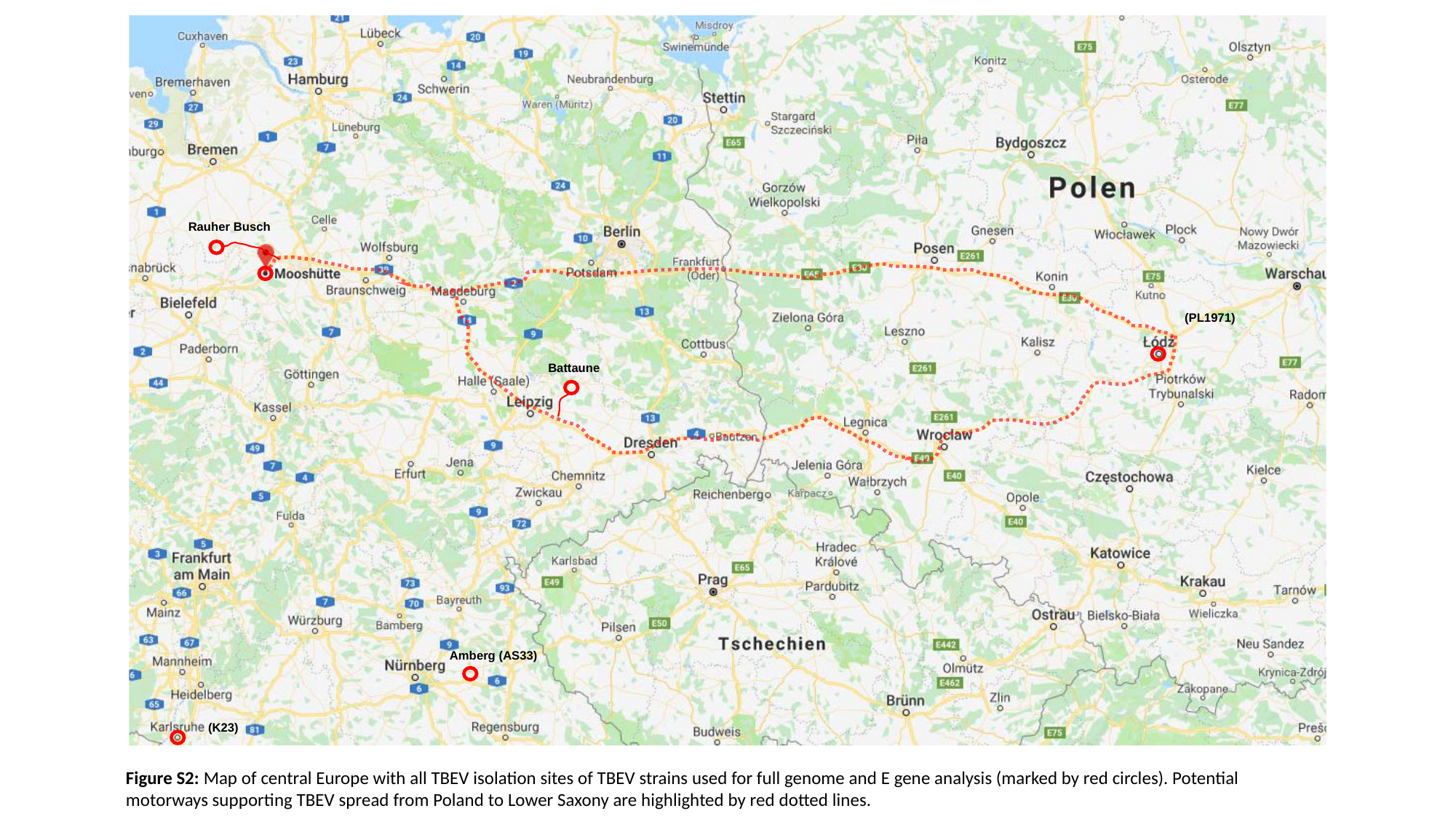

Rauher Busch
Battaune
Amberg (AS33)
 (K23)
 (PL1971)
Figure S2: Map of central Europe with all TBEV isolation sites of TBEV strains used for full genome and E gene analysis (marked by red circles). Potential motorways supporting TBEV spread from Poland to Lower Saxony are highlighted by red dotted lines.

## Slide 3
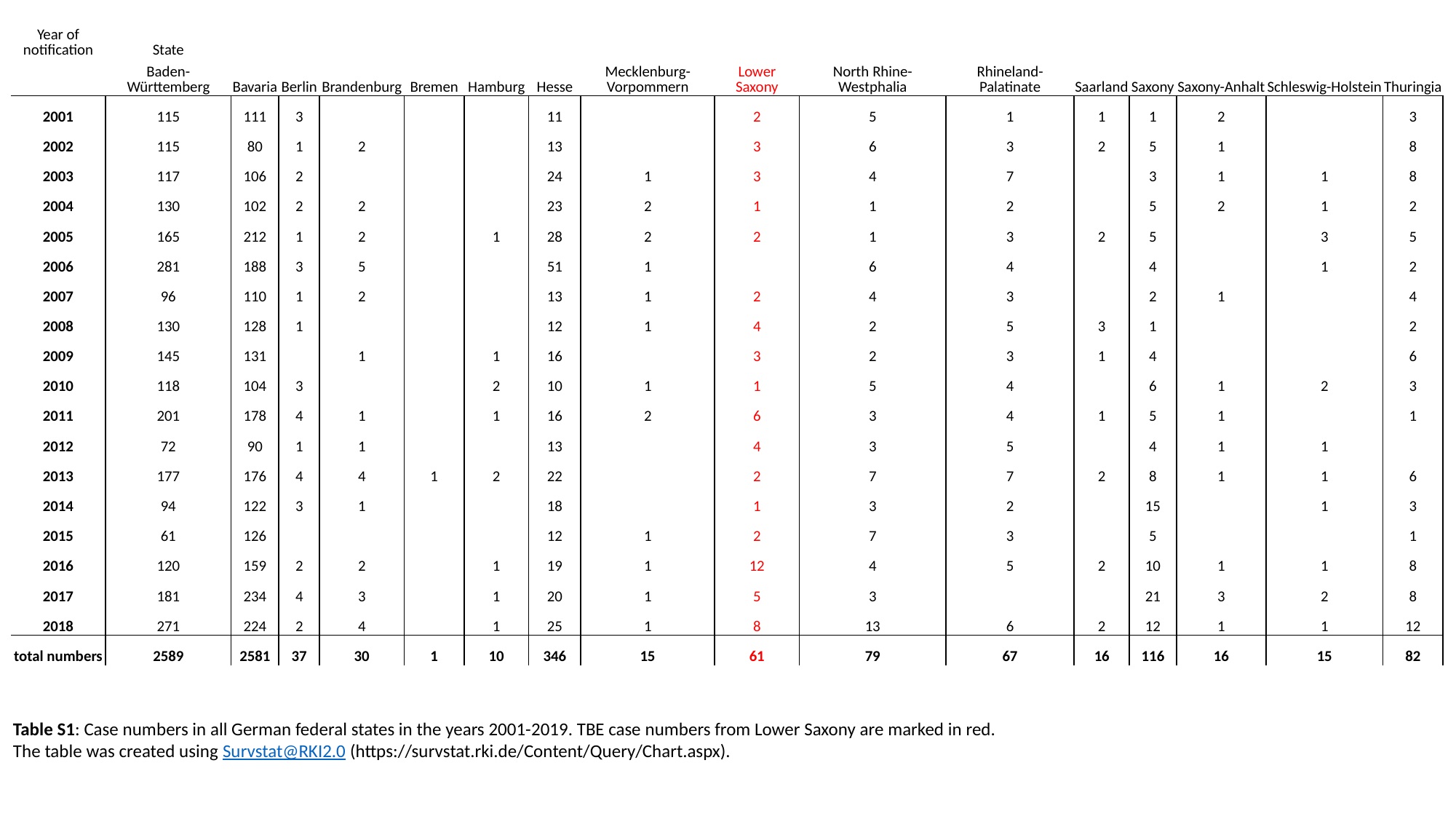

| Year of notification | State | | | | | | | | | | | | | | | |
| --- | --- | --- | --- | --- | --- | --- | --- | --- | --- | --- | --- | --- | --- | --- | --- | --- |
| | Baden-Württemberg | Bavaria | Berlin | Brandenburg | Bremen | Hamburg | Hesse | Mecklenburg-Vorpommern | Lower Saxony | North Rhine-Westphalia | Rhineland-Palatinate | Saarland | Saxony | Saxony-Anhalt | Schleswig-Holstein | Thuringia |
| 2001 | 115 | 111 | 3 | | | | 11 | | 2 | 5 | 1 | 1 | 1 | 2 | | 3 |
| 2002 | 115 | 80 | 1 | 2 | | | 13 | | 3 | 6 | 3 | 2 | 5 | 1 | | 8 |
| 2003 | 117 | 106 | 2 | | | | 24 | 1 | 3 | 4 | 7 | | 3 | 1 | 1 | 8 |
| 2004 | 130 | 102 | 2 | 2 | | | 23 | 2 | 1 | 1 | 2 | | 5 | 2 | 1 | 2 |
| 2005 | 165 | 212 | 1 | 2 | | 1 | 28 | 2 | 2 | 1 | 3 | 2 | 5 | | 3 | 5 |
| 2006 | 281 | 188 | 3 | 5 | | | 51 | 1 | | 6 | 4 | | 4 | | 1 | 2 |
| 2007 | 96 | 110 | 1 | 2 | | | 13 | 1 | 2 | 4 | 3 | | 2 | 1 | | 4 |
| 2008 | 130 | 128 | 1 | | | | 12 | 1 | 4 | 2 | 5 | 3 | 1 | | | 2 |
| 2009 | 145 | 131 | | 1 | | 1 | 16 | | 3 | 2 | 3 | 1 | 4 | | | 6 |
| 2010 | 118 | 104 | 3 | | | 2 | 10 | 1 | 1 | 5 | 4 | | 6 | 1 | 2 | 3 |
| 2011 | 201 | 178 | 4 | 1 | | 1 | 16 | 2 | 6 | 3 | 4 | 1 | 5 | 1 | | 1 |
| 2012 | 72 | 90 | 1 | 1 | | | 13 | | 4 | 3 | 5 | | 4 | 1 | 1 | |
| 2013 | 177 | 176 | 4 | 4 | 1 | 2 | 22 | | 2 | 7 | 7 | 2 | 8 | 1 | 1 | 6 |
| 2014 | 94 | 122 | 3 | 1 | | | 18 | | 1 | 3 | 2 | | 15 | | 1 | 3 |
| 2015 | 61 | 126 | | | | | 12 | 1 | 2 | 7 | 3 | | 5 | | | 1 |
| 2016 | 120 | 159 | 2 | 2 | | 1 | 19 | 1 | 12 | 4 | 5 | 2 | 10 | 1 | 1 | 8 |
| 2017 | 181 | 234 | 4 | 3 | | 1 | 20 | 1 | 5 | 3 | | | 21 | 3 | 2 | 8 |
| 2018 | 271 | 224 | 2 | 4 | | 1 | 25 | 1 | 8 | 13 | 6 | 2 | 12 | 1 | 1 | 12 |
| total numbers | 2589 | 2581 | 37 | 30 | 1 | 10 | 346 | 15 | 61 | 79 | 67 | 16 | 116 | 16 | 15 | 82 |
Table S1: Case numbers in all German federal states in the years 2001-2019. TBE case numbers from Lower Saxony are marked in red.
The table was created using Survstat@RKI2.0 (https://survstat.rki.de/Content/Query/Chart.aspx).
