# Supplementary material for: First Isolation and Phylogenetic Analyses of Tick-Borne Encephalitis Virus in Lower Saxony, Germany
Source: Viruses. 2019 May 21;11(5):462. doi: 10.3390/v11050462 (PMC6563265; doi:10.3390/v11050462)
Supplement: Supplementary file 1 [file viruses-11-00462-s001.zip › Table S2.docx]

Table S2: Sequence comparison of the isolates TBEV-LS-Rauher Busch P19 and TBEV-LS-Barsinghausen/Mooshuette HB IF06 8033. All nucleotide differences are given and all non-synonymous mutations are marked red.

|  | **Rauher Busch** | | **Barsinghausen/Mooshuette** | |
| --- | --- | --- | --- | --- |
| **Base No.** | **nt** | **aa** | **nt** | **aa** |
| 246 | T | V | G | V |
| 1251 | G | M | A | I |
| 2058 | A | T | C | T |
| 2568 | A | A | G | A |
| 2580 | G | A | A | A |
| 2868 | T | D | C | D |
| 3282 | A | T | G | T |
| 3895 | C | H | T | Y |
| 4074 | A | R | G | R |
| 4326 | C | H | T | H |
| 4327 | C | L | T | L |
| 4665 | T | A | C | A |
| 4845 | G | L | T | L |
| 5301 | A | P | T | P |
| 5343 | T | A | C | A |
| 5364 | T | S | C | S |
| 5795 | G | R | A | K |
| 5879 | T | I | C | T |
| 6057 | G | E | A | E |
| 6111 | G | L | A | L |
| 6198 | T | A | C | A |
| 6264 | A | K | G | K |
| 6804 | T | T | C | T |
| 7137 | C | H | T | H |
| 7318 | T | C | A | S |
| 7365 | A | E | G | E |
| 7404 | C | L | T | L |
| 7835 | G | R | A | K |
| 8684-8685 | AA | E | GG | G |
| 9098 | A | K | G | R |
| 9271 | G | E | A | K |
| 9339 | C | G | T | G |
| 9417 | A | E | G | E |
| 9906 | C | T | T | T |
| 10029 | A | I | G | M |
| 10137 | A | V | G | V |
